# Finite Codimension Stability of Invariant Surfaces

**DOI:** 10.1007/s00220-026-05764-5

**Published:** 2026-10-26

**Authors:** Giovanni Forni

**Affiliations:** 1 Department of Mathematics, University of Maryland, College Park, MD USA; 2 CY Cergy Paris Université, Cergy-Pontoise, France

## Abstract

Following recent work of Alazard and Shao [AlSh] on applications of para-differential calculus to smooth conjugacy and stability problems for Hamiltonian systems, we prove finite codimension stability of invariant surfaces (in finite differentiability classes) of flat geodesic flows on translation surfaces. The result is also based on work of the author [F97, F21] on the cohomological equation for translation flows.

## Introduction

The billiard in a completely integrable rational polygon, as well as the geodesic flow on a flat torus, are basic examples of integrable Hamiltonian systems: their phase space is entirely foliated by invariant 2-dimensional tori on which the flow is linear. In this context the KAM theory implies that, under sufficiently small smooth perturbations of the Hamiltonian, a positive measure set of invariant tori persists and therefore the perturbed system is not ergodic.

In this paper, we prove an analogous result for a class of pseudo-integrable systems: (non-integrable) billiards in rational polygons and, more generally, geodesic flows for the flat metric on translation surfaces. The phase space of these systems is foliated by invariant surfaces of higher genus (in the non-integrable case) and it is natural to ask whether any of such surfaces persists under small smooth perturbations of the Hamiltonian.

With this problem in mind the author proved a result [F97] on the linearized problem, that is, on the so-called cohomological equation for translation flows on higher genus surfaces. This work found that, contrary to the case of (Diophantine) linear toral flows, the Lie derivative operator for translation flows on higher genus surface has range of finite codimension in every finite differentiability class, and of infinite codimension in the space of infinitely differentiable functions.

In addition, the obstructions to the existence of solutions depend on the specific translation flow considered, while in the case of the torus the Lie derivative operator has range of codimension one, transverse to the space of constant functions, and the only obstruction, the mean, is independent of the linear flow.

As a consequence of this result, it was natural to conjecture, transposing the results on the linearized problem to the non-linear problems, that typical translation flows would be stable with finite codimension in any finite differentiability class under smooth perturbations, and that the typical invariant surfaces of pseudo-integrable systems would be similarly stable with finite codimension, hence there would exists a finite codimension family of non-ergodic perturbations.

Unfortunately the presence of distributional obstructions of growing Sobolev order creates serious difficulty in the application of the KAM or Nash-Moser iteration method, which we have not been overcome to date.

These difficulties are caused by the feature of the Nash-Moser iteration of letting the high norms grow (while controlling the decay of low norms), as a consequence of the application of smoothing operators at each step of the iteration. Therefore the values of the higher order obstructions on the data of the linearized equation may blow up in the iteration process which therefore may fail to converge.

For the conjugacy problem, S. Marmi, P. Moussa and J.-C.Yoccoz [MMY12] were able to bypass the difficulty by a method inspired to M. Herman’s fixed point solution of the conjugacy problem for typical (Roth-type) circle rotations  [He85], based on a “Schwarzian derivative trick”.

The work [MMY12] was made possible by improved regularity estimates for solutions of the cohomological equation for Interval Exchange Transformations (and translation flows) derived in [MMY05] by a dynamical approach completely different from the harmonic analysis methods of [F97].

In fact, Herman’s method requires a loss of at most 2- derivatives for the solutions of the cohomological equation, a result which is out of the reach of the method of [F97] (see also [F21]). In [MMY05] the loss of derivatives is (at most) 1+BV, while in [F97] is at best 3+ (and this result is only achieved in [F21]).

For low regularity conjugacies (that is, for conjugacies of class $C^1$) and for the global conjugacy problem, S. Ghazouani [Gh21] and S. Ghazouani and C. Ulcigrai [GU23] have developed a renormalization approach. In [Gh21] a $C^1$ conjugacy result is proved for Interval Exchange Transformations of periodic type, while in [GU23] (followed by a refined result [GU25] on the regularity of the conjugacy), the authors prove a rigidity result, that is, a higher genus version of Herman’s global linearization theorem for circle diffeomorphisms [He79].

The analogous question on the stability of invariant (higher genus) surfaces under smooth perturbations has so far remained open.

In this paper we follow the para-differential calculus approach of T. Alazard and C. Shao [AlSh] and give a proof of finite codimension stability in the Sobolev scale of a typical invariant surface. Indeed, the para-differential approach appears especially powerful to treat non-linear problems with finite codimension, increasing with the regularity class or with infinite codimension, for which a KAM approach is problematic and has not so far been implemented. We believe that the para-differential method could lead to another proof of the linearization result [MMY12], possibly with somewhat stronger regularity assumptions, but under “Diophantine” conditions weaker than the Roth-type condition assumed there.

Our main result is the following

### Theorem 1.1

Let (M,ω) any translation surface. For almost all $\xi \in P^1({ \mathbb {R}}^2)$, the invariant 2-dimensional surface $M_\xi$ in a given energy level for the flat geodesic flow given by all tangent vector parallel to ξ and fixed norm, is stable with finite codimension under smooth perturbations in the the following sense.

There exists $s_0>0$ such that for all $s>s_0$ there exists a local subvariety $\mathcal {H}_s(\xi )$ (a priori dependent on $\xi \in P^1({ \mathbb {R}}^2)$) of finite codimension $h_s\in { \mathbb {N}}$ of the space of Hamiltonians such that for all Hamiltonians sufficiently close to the Hamiltonian $H_0$ of the flat geodesic flow in the Sobolev space $H^s(M)$ and equal to $H_0$ near the set $\Sigma :=\{\omega =0\}$, the Hamiltonian flow of *H* has an invariant surface $M^H_\xi$ of genus equal to the genus of *M*. The invariant surface $M^H_\xi$ is an $H^t(M)$-graph over $M_\xi$ for $t<s-s_0$ and the Hamiltonian flow of *H* on $M^H_\xi$ is $H^t(M)$-conjugated to the translation flow given by the restriction of the flat geodesic flow to $M_\xi$. The codimension $h_s$ of the subvariety $\mathcal {H}_s(\xi )$ grows linearly in $s>s_0$ and in the genus of the surface *M* and the cardinality of Σ.

From Theorem 1.1 we derive the following result for billards in rational polygons.

### Corollary 1.2

For any rational polygon *P* and for almost all $\xi \in P^1({ \mathbb {R}}^2)$, the invariant 2-dimensional surface $M_\xi$ for the billiard flow in *P*, endowed with the flat metric $R_0$, in direction ξ and in a given energy level, is stable with finite codimension under smooth perturbations in the the following sense. There exists $s_0>0$ such that for all $s>s_0$ there exists a local subvariety $\mathcal {K}_s(\xi )$ (a priori dependent on $\xi \in P^1({ \mathbb {R}}^2)$) of finite codimension $k_s\in { \mathbb {N}}$ of the space of metric on *P* sufficiently close to the flat metric $R_0$ in the Sobolev space $H^s(M)$, and equal to the flat metric near its corners, such that the billiard flow in *P* with respect to any metric $R\in \mathcal {K}_s$ has an invariant surface $M^R_\xi$ of genus equal to the genus of the unfolding of *P*. The invariant surface $M^R_\xi$ is an $H^t(M)$-graph over $M_\xi$ for $t<s-s_0$ and the billiard flow for *P*, endowed with the metric *R*, on $M^R_\xi$ is $H^t(M)$-conjugated to the translation flow given by the restriction of the flat metric billiard flow to $M_\xi$. The codimension $k_s$ of the subvariety $\mathcal {K}_s(\xi )$ grows linearly in $s>s_0$ and in the genus and the cardinality of the set of conical singularities of the translation surface $M_P$ unfolding of *P*.

## Sobolev Spaces and Para-Differential Operators

In this section we recall the definition of the natural (weighted) Sobolev spaces on translation surfaces (see [F97, F21]), and extend the para-differential formalism to translation surfaces (following [AlSh]).

Let (M,ω) be a translation surface. Let $L^2(M, \omega )$ denote the space of square-integrable functions with respect to the area form $dA_\omega = (i/2) \omega \wedge \bar{\omega }$. Let *X* and *Y* denote the horizontal and vertical vector fields defined by the conditions

$$\begin{aligned} \imath _X {\operatorname {Re} }(\omega ) = \imath _Y {\operatorname {Im} }(\omega ) = 1, \quad \text { and } \quad \imath _X {\operatorname {Im} }(\omega ) = \imath _Y {\operatorname {Re} }(\omega ) = 0. \end{aligned}$$

With respect to a canonical coordinate *z* centered at a regular point of (M,ω), that is, a coordinate such that ω=dz, we have $dA_\omega = dx \wedge dy$ and

$$\begin{aligned} X= \frac{\partial }{\partial x} \quad \text { and } \quad Y= \frac{\partial }{\partial y} . \end{aligned}$$

With respect to a canonical coordinate *z* centered at a cone point of (M,ω) of angle 2π(k+1), that is, a coordinate such that $\omega = z^k dz$, we have $dA_\omega =\vert z \vert ^{2k} dx \wedge dy$ and

$$\begin{aligned} X= \vert z \vert ^{-2k} \Big ( {\operatorname {Re} }(z^k) \frac{\partial }{\partial x} - {\operatorname {Im} }(z^k) \frac{\partial }{\partial y}\Big ) \quad \text { and } \quad Y= \vert z \vert ^{-2k} \Big ( {\operatorname {Im} }(z^k) \frac{\partial }{\partial x} + {\operatorname {Re} }(z^k) \frac{\partial }{\partial y}\Big ) . \end{aligned}$$

In other terms, for any p∈M a cone point of angle 2π(k+1), the map $\pi _k: U(p) \rightarrow D \subset {\mathbb {C}}$ defined on a neighbourhood U(p)⊂M such that $U(p) \cap \Sigma =\{p\}$ with respect to a canonical coordinate $z: U(p) \rightarrow D \subset {\mathbb {C}}$ as

$$\begin{aligned} \pi _k (z) = \frac{ z^{k+1} }{k+1} , \quad \text { for all } z\in U(p), \end{aligned}$$

is a (k+1)-fold branched cover of a neighborhood *D* of $0\in {\mathbb {C}}$ such that $\pi _k^*(dz) = \omega \vert U(p)$ and

$$\begin{aligned} (\pi _k)_*(X) = \frac{\partial }{\partial x} \quad \text { and } \quad (\pi _k)_*(Y) = \frac{\partial }{\partial y} . \end{aligned}$$

The (weighted) Sobolev spaces $H^s_\omega (M)$ (for s≥0) can be defined as the subspaces of $f\in L^2(M, \omega )$ such that $f \in H^s_{loc} (M{\setminus } \Sigma )$ and for every cone point $p\in \Sigma$ (of angle 2π(k+1)) there exists a function $F \in H^s(D)$ such that $f = (\pi _k)^*(F)$. In short, for any translation atlas $\mathcal {U}:=\{(U, \pi _U)\}$ on *M* (composed of charts given by canonical coordinates)

$$\begin{aligned} f \in H^s_\omega (M) \Longleftrightarrow f \vert U \in (\pi _U)^*( H^s ( \pi _U (U) ) \quad \text { for all } (U, \pi _U) \in \mathcal {U}. \end{aligned}$$

The norm on the space $H^s_\omega (M)$ can be defined for $s \in { \mathbb {N}}$ as follows:

$$\begin{aligned} \Vert f \Vert _{H^s_\omega (M)}^2 = \sum _{\alpha +\beta \le s} \Vert X^\alpha Y^\beta \Vert ^2_{L^2(M, \omega )} , \quad \text { for all } f \in H^\infty _\omega (M). \end{aligned}$$

Another possible definition of Sobolev norms on translation surfaces is in terms of fractional powers of the Friedrichs Laplacian. Let $\Delta _F$ denote the Friedrichs extension of the flat Laplacian with domain $H^\infty _\omega (M)$ and let $\{\lambda _n\}_{n\in { \mathbb {N}}}$ denote the sequence of eigenvalues of the negative of the Friedrichs Laplacian $-\Delta _F$ and let $\{ e_n\}$ a corresponding orthonormal system of eigefunctions.

The Friedrichs (weighted) Sobolev norms (for all $s \in { \mathbb {R}}$) are

$$\begin{aligned} \Vert f \Vert _{\bar{H}^s_\omega (M)}^2 = \sum _{n\in { \mathbb {N}}} (1+ \lambda _n)^s \vert \langle f, e_n\rangle \vert ^2 , \quad \text { for all } f \in H^\infty _\omega (M). \end{aligned}$$

By definition the Friedrichs Sobolev norms are interpolation norms.

The fractional (weighted) Sobolev norms can then be defined as follows: for any $s= k + \sigma \ge 0$ with $k\in { \mathbb {N}}$ and $\sigma \in [0, 1)$, we define, for all $f \in H^\infty _\omega (M)$,

$$\begin{aligned} \Vert f \Vert _{H^s_\omega (M)}^2 = \Vert f \Vert _{H^{k}_\omega (M)}^2 + \sum _{\alpha + \beta =k} \Vert X^\alpha Y^\beta f \Vert _{\bar{H}^{\sigma }_\omega (M)}^2 + \Vert Y^\alpha X^\beta f \Vert _{\bar{H}^{\sigma }_\omega (M)}^2 . \end{aligned}$$

We have the following comparison result between the weighted Sobolev norms, the Friedrichs weighted Sobolev norms and the standard Sobolev norms:

### Lemma 2.1

([F21], Lemma 2.11). The following continuous embedding and isomorphisms of Banach spaces hold:

- $$H^s(M) \subset H^s_\omega (M) \equiv \bar{H}^s_\omega (M)$$,       for 0≤s<1;
- $$H^s(M) \equiv H^s_\omega (M) \equiv \bar{H}^s_\omega (M)$$,       for s=1;
- $$H^s_\omega (M) \subset \bar{H}^s_\omega (M) \subset H^s(M)$$,       for s>1 .

For s∈[0,1] the space $H^s(M)$ is dense in $H^s_\omega (M)$ and, for s>1, the closure of $H^s_\omega (M)$ in $\bar{H}^s_\omega (M)$ or $H^s(M)$ has finite codimension.

The weighted Sobolev spaces $H^{-s}_\omega (M)$ with negative exponents are defined as the dual spaces of the spaces $H^s_\omega (M)$ for all s>0.

Para-differential operators on Euclidean spaces were introduced by M. Bony in [Bo81] (see also [Me08]). Para-differential operators can be generalized to smooth compact manifolds working in local coordinates.

A recent detailed introduction of para-differential calculus on compact manifolds can be found in [BGdP21], §2, in [Del15], §3, in Chap. 6 of [Sh22] (and to some extent earlier in [Tay91], Chap. 3, [Tay11], Chap 13. 10).

Para-differential operators can therefore be extended to translation surfaces by defining para-differential operators locally with respect to canonical charts. Since all the results are local and weighted Sobolev spaces are defined in terms of canonical charts, they generalize to our context first for functions in $H^\infty _\omega (M)$, then by continuity to functions in the spaces $H^s_\omega (M)$. In particular we can define para-products $Op(a):=T_a$ for functions $a \in L^\infty (M)$.

We have the following results (see [AlSh], Props. 2.1 −2.3 and Prop. 3.5):

### Proposition 2.2

(Continuity of para-product operators). If $a\in L^\infty (M)$, then $T_a$ is a bounded linear operator from $H^s_\omega (M)$ to itself, and in fact there exists a constant $C_s>0$ such that

$$\begin{aligned} \Vert T_a \Vert _{\mathcal {L}( H^s_\omega (M), H^s_\omega (M)) } \le C_s \Vert a \Vert _{L^\infty (M)}. \end{aligned}$$

Let now $C^r_\omega (M)$ denote the space of functions which belong to the Zygmund (or Lipschitz) space $C^r_*$ locally with respect to canonical coordinates on (M,ω).

### Proposition 2.3

(Composition of para-product operators). If $a, b\in C^r_\omega (M)$, then $T_{ab} -T_a T_b$ is a bounded linear operator from $H^s_\omega (M)$ to $H^{s+r}_\omega (M)$, and in fact there exists a constant $C_{r,s}>0$ such that

$$\begin{aligned} \Vert T_{ab} -T_a T_b \Vert _{\mathcal {L}( H^s_\omega (M), H^{s+r}_\omega (M)) } \le C_{r,s} \Vert a \Vert _{C^r_\omega } \Vert b \Vert _{C^r_\omega }. \end{aligned}$$

### Proposition 2.4

(Para-linearization). Let s>1 and let $N_s\in { \mathbb {N}}$ denote the smallest integer such that $N_s > 2s-1$. For any functions $u \in H^s_\omega (M, { \mathbb {R}}^2)$ and $F:= F(x,u) \in C^{N_s+3}_\omega (M \times { \mathbb {R}}^2 )$, the following para-linearization formula holds:

$$\begin{aligned} F(x,u)-F(x,0)= Op({ \frac{\partial F(x,u)}{ \partial u}} ) u + \mathcal {R}_{PL} (F(x,\cdot ),u)u \in H^s_\omega (M) +H^{2s-1} _\omega (M), \end{aligned}$$

where $\mathcal {R}_{PL} (F(x, \cdot ),u)u$ is a bounded linear operator from $H^s_\omega (M)$ to $H^{2s-1}_\omega (M)$ such that for a constant $C'_s >0$

$$\begin{aligned} \Vert \mathcal {R}_{PL} (F(x,\cdot ),u) \Vert _{\mathcal {L}(H^{s}_\omega (M), H^{2s-1}_\omega (M) )} \le C'_s \, \Vert F \Vert _{C^{N_s+3}_\omega (M \times { \mathbb {R}}^2 )} (1+\Vert u \Vert _{ H^s_\omega (M)}) . \end{aligned}$$

Moreover, the operators $Op({ \frac{\partial F(x,u)}{ \partial u}} ) \in \mathcal {L}(H^{s}_\omega (M), H^{s}_\omega (M))$ and $\mathcal {R}_{PL} (F(x,\cdot ),u) \in \mathcal {L}(H^{s}_\omega (M), H^{2\,s-1}_\omega (M))$ are continuously differentiable in $u\in H^s_\omega (M)$ with respect to the operator norms.

##  Geodesic Flow on Translation Surfaces

The Hamiltonian of the flat geodesic flow on a translation surface (M,ω) has the form on $M\setminus \Sigma$ in canonical coordinates:

$$\begin{aligned} H_0(x, \xi ) = \frac{\xi _1^2 + \xi _2^2}{2} \quad \text { for all } (x, \xi ) \in M\setminus \Sigma \times { \mathbb {R}}^2. \end{aligned}$$

The coordinate-free expression of the Hamiltonian is

$$\begin{aligned} H_0(x, v) = \frac{1}{2} \vert \omega _x (v ) \vert ^2 , \quad \text { for all } (x,v) \in TM. \end{aligned}$$

We will consider a Hamiltonian function

$$\begin{aligned} H(x, v) = \frac{1}{2} \vert \omega _x (v ) \vert ^2 + f(x, v) , \quad \text { for all } (x,v) \in TM. \end{aligned}$$

with *f* a smooth function vanishing on a neighborhood of $TM \vert \Sigma$ (in fact, it is enough to assume vanishing at $TM \vert \Sigma$ with sufficiently high order).

The tangent bundle $TM \vert \Sigma$ can be trivialized over $M\setminus \Sigma$ since the bundle has never vanishing sections *X* and *Y*, hence

$$\begin{aligned} v = X_\xi := \xi _1 X_1+ \xi _2 X_2 , \end{aligned}$$

and in the same coordinates

$$\begin{aligned} H(x, \xi ) = \frac{\xi _1^2 + \xi _2^2}{2} + f(x, \xi ). \end{aligned}$$

The Hamiltonian vector field $X_H$ has the form

$$\begin{aligned} X_H (x,\xi )&= \frac{\partial H}{\partial \xi _1} X_1 + \frac{\partial H}{\partial \xi _2} X_2 - X_1 H \frac{\partial }{\partial \xi _1} - X_2H \frac{\partial }{\partial \xi _2} \\&= \xi _1 X_1 + \xi _2 X_2 + \frac{\partial f}{\partial \xi _1}(x,\xi ) X_1 + \frac{\partial f}{\partial \xi _2}(x,\xi ) X_2 \\&\quad - X_1 f (x,\xi ) \frac{\partial }{\partial \xi _1} - X_2 f (x,\xi ) \frac{\partial }{\partial \xi _2}\,. \end{aligned}$$

The equation of an invariant surface is of the form

1 $$\begin{aligned} \mathcal {F}_\xi (H, u) := X_H \circ u - X_\xi (u) =0 \,. \end{aligned}$$

with $u: M \rightarrow M\times { \mathbb {R}}^2$, so that the invariant surface is $u(M) \subset M\times { \mathbb {R}}^2$.

We proceed to a (standard) computation of the differential $D_u \mathcal {F}_\xi (H, u)$. Let

$$\begin{aligned} A[u] = \begin{pmatrix} D_X \nabla _\xi H (u) & D_\xi \nabla _\xi H (u)\\ - D_X \nabla _X H (u) & - D_\xi \nabla _X H (u) \end{pmatrix} \in M_{4\times 4} ({ \mathbb {R}}). \end{aligned}$$

so that

$$\begin{aligned} D_u \mathcal {F}_\xi (H, u) (v) = A[u] (v) - X_\xi (v) . \end{aligned}$$

Following [AlSh, LGJV05], we introduce

$$\begin{aligned} N[u] = (Du^t \cdot Du)^{-1} \in M_{2\times 2} ({ \mathbb {R}}) \quad \text { and } \quad M[u] = \begin{pmatrix} Du&(JDu) \cdot N[u] \end{pmatrix} \in M_{4\times 4}({ \mathbb {R}}) . \end{aligned}$$

In the above formulas *Du* denotes the differential of $u = (u_1, u_2): M \rightarrow M \times { \mathbb {R}}^2$, with respect to the bases $\{X_1, X_2\}$ of *TM* and $\{ \partial / \partial \xi _1, \partial / \partial \xi _2\}$, as a column vector:

$$\begin{aligned} Du = \begin{pmatrix} Du_1 \\ D u_2 \end{pmatrix} \in M_{4, 2} ({ \mathbb {R}}), \quad JDu= \begin{pmatrix} 0&I_2 \textrm{i}_2&0 \end{pmatrix} \begin{pmatrix} Du_1 \\ D u_2 \end{pmatrix} = \begin{pmatrix} Du_2 \\ -D u_1 \end{pmatrix} \in M_{4, 2} ({ \mathbb {R}}). \end{aligned}$$

Since *u* is by hypothesis close to $u_0$, defined as $u_0(x)= (x, \xi )$ for $(x, \xi ) \in M \times { \mathbb {R}}^2$, it follows that *N*[*u*] is close to the identity, and *M*[*u*], $M[u]^{-1}$ are close to $\text {diag}(I_2, -I_2)$ (with an error uniformly bounded in terms of the uniform norm of *Du*).

Since the translation structure on *M* (of genus g≥2) has cone points at a finite set Σ, the space of smooth maps $u: M \rightarrow M \times { \mathbb {R}}^2$ is not locally a vector space. However, the finite codimensional subspace determined by the condition that the restriction

$$\begin{aligned} u_1 \vert \Sigma = u_{0,1} \vert \Sigma = \text {Id}_\Sigma \, \end{aligned}$$

can be locally identified with a ball (with sufficiently small radius) of functions with values in ${ \mathbb {R}}^2 \times { \mathbb {R}}^2$ which vanish at Σ. Indeed, for such functions we may have (in terms of the flat distance $d_\omega$ on the translation surface (M,ω)):

$$\begin{aligned} \text {d}_\omega ( u(x), x ) < \text {d}_\omega ( x, \Sigma ), \quad \text { for all } x \in M\setminus \Sigma . \end{aligned}$$

The linearization of the equation (1) is a cohomological equation for the translation vector field $X_\xi$. Cohomological equations on translation surfaces were investigated in [F97, F21], and [MMY05, MY16] for Interval Exchange Transformations (IET’s), which appear as return maps of translation flows to transverse intervals. All of the above paper, except [F97] hold for almost all translation surfaces, in fact under a precise Roth-type full measure condition on the IET. It was proved in [CE15] on the basis of the Magic Wand Theorem of Eskin, Mirzakhani and Mohammadi  [EM18, EMM15] and subsequent results of S. Filip [Fil16], that the Roth-type condition in fact holds for every translation surface in almost all directions.

We state below the simplest form of such results, going back to [F97].

For all s≥0, and for almost all $\xi \in { \mathbb {R}}^2$ let $\mathcal {I}^s_\xi (M) \subset H^{-s}_\omega (M)$ denote the space of invariant distributions for the vector field $X_\xi$ on *M*, that is,

$$\begin{aligned} \mathcal {I}^s_\xi (M):= \{ D \in H^{-s}_\omega (M) \vert X_\xi D =0 \}. \end{aligned}$$

### Theorem 3.1

[F97]. There exists $s_0 >0$ such that, for all $s >s_0$ and for almost all $\xi \in { \mathbb {R}}^2$ the cohomological equation $X_\xi u =f$ has a solution under the following conditions:

- there exists a constant $C_{s}(\xi ) >0$ such that if $f \in H^s_\omega (M)$ has zero average with respect to the area form on (M,ω) then there exists a distributional solution $u \in H^{-s_0}_\omega (M)$ such that
  $$\begin{aligned} \Vert u \Vert _{H^{-s_0}_\omega (M)} \le C_s(\xi ) \Vert f \Vert _{H^{s}_\omega (M)} ; \end{aligned}$$
- for all $0\le t <s-s_0$ and there exists a constant $C_{s,t}(\xi ) >0$ such that if $f \in H^s_\omega (M)$ belongs to the kernel $\text {Ker} \Big (\mathcal {I}^s_\xi (M) \Big )$ of the space of invariant distributions, which is finite dimensional, then there exists a unique zero-average solution $u\in H^t_\omega (M)$ and the following estimate holds:
  $$\begin{aligned} \Vert u \Vert _{H^{t}_\omega (M)} \le C_{s,t} (\xi ) \Vert f \Vert _{H^{s}_\omega (M)} ; \end{aligned}$$

It is then immediate to derive the existence of solutions vanishing (at any finite order) at Σ under a finite number of additional independent distributional conditions.

### Corollary 3.2

For any $k\in { \mathbb {N}}$, there exists $s_k >0$ such that, for all $s >s_k$ and for almost all $\xi \in { \mathbb {R}}^2$, there exists a finite dimensional space $\mathcal {I}^{s,k}_\xi (M) \subset H^{-s}_\omega (M)$ such that the cohomological equation $X_\xi u =f$ has a solution vanishing at order *k* on the finite set Σ under the condition that $f \in \text {Ker}\Big ( \mathcal {I}^{s,k}_\xi (M) \Big ) \subset H^{s}_\omega (M)$ .

### Proof

By Theorem 3.1, for all $s>s_0$ and for almost all $\xi \in { \mathbb {R}}^2$, there exists a Green operator $G^s_\xi : \text {Ker}\Big ( \mathcal {I}^{s}_\xi (M) \Big ) \rightarrow H^t_\omega (M)$ for $t<s-s_0$ (with values in the subspace of zero average functions). The condition of vanishing at Σ at order *k* is given by a finite number of distributions supported at Σ, which are derivatives of Dirac masses at Σ, with Sobolev order up to k+1 (by the Sobolev embedding theorem). Let us assume then that $s_k> s_0 + k +1$. By Theorem 3.1 the compositions $\delta _\Sigma ^{(j)} \circ G_\xi ^s$, of derivatives $\delta _\Sigma ^{(j)}$ of order j≤k of Dirac masses at Σ with the Green operator $G^s_\xi$, give well-defined distributions on $H^{s_k}_\omega (M)$, since $s_k -s_0 > k+1$.

By definition, if $f\in H^s_\omega (M)$ with $s>s_k$ belongs to the kernel of $\mathcal {I}^{s}_\xi (M)$ and to that of all the additional distributions $\delta _\Sigma ^{(j)} \circ G_\xi ^s$, then the unique zero-average solution $u\in H^t_\omega (M)$ of the equation $X_\xi u=f$ vanishes at order *k* on Σ. □

It is well known that invariant submanifolds of Hamiltonian flows should be expected to be Lagrangian. In our context for any differentiable map $u: M \rightarrow { \mathbb {R}}^2$, the function

$$\begin{aligned} L[u]= (Du)^t J Du , \end{aligned}$$

measures how for *u* is from being a Lagrangian embedding. In fact L[u]=0 if and only if the pull-back under *u* of the standard symplectic form on $M \times { \mathbb {R}}^2$ vanishes.

In addition L[u]=0 whenever $\mathcal {F}_\xi (H, u)=0$ (*u* is an invariant section) and the Sobolev norms of *L*[*u*] can be bounded in terms of those of $\mathcal {F}_\xi (H, u)$, as in the following result.

### Lemma 3.3

(see [LGJV05, Lemma 19]). If the translation flow in the direction $\xi \in { \mathbb {R}}^2\setminus \{0\}$ is (quasi)-minimal on *M*, then the function *L*[*u*] depends linearly on $\mathcal {F}_\xi (H, u)$ for all continuously differentiable $u:M \rightarrow { \mathbb {R}}^2$. In fact, we have the formula

$$\begin{aligned} X_\xi L[u] = - \Big [D \mathcal {F}_\xi (H, u)^t J Du + (Du)^t J D\mathcal {F}_\xi (H, u) \Big ] . \end{aligned}$$

As a consequence, for almost all $\xi \in { \mathbb {R}}^2$, for every $s>s_0$ and any $t<s-s_0$ there exists $C_{s,t}(\xi ) >0$ such that, whenever $u \in H^{s+2}_\omega (M)$, we have

$$\begin{aligned} \Vert L[u] \Vert _{H^t_\omega (M)} \le C_{s,t}(\xi ) \Vert Du \Vert _{H^s_\omega (M)} \Vert D\mathcal {F}_\xi (H, u) \Vert _{H^s_\omega (M)} . \end{aligned}$$

### Proof

Since by definition $$\mathcal {F}_\xi (H, u) = X_H \circ u - X_\xi u$$ and $$D (X_H \circ u) = A[u] Du$$, and by the Hamiltonian character of the equations $$ A[u]^t J + J A[u] =0$$, we can compute as follows:

$$\begin{aligned} X_\xi L[u]&= (D X_\xi u)^t J Du + (Du)^t J D X_\xi u \\ &= \Big [D(X_H\circ u - \mathcal {F}_\xi (H, u) )\Big ]^t J Du + (Du)^t J D \Big [ (X_H \circ u -\mathcal {F}_\xi (H, u)\Big ] \\ &= (Du)^t \Big ( A[u]^t J + J A[u] \Big ) Du - D \mathcal {F}_\xi (H, u)^t J Du - (Du)^t J D\mathcal {F}_\xi (H, u) \Big ] \\ &= - \Big [D \mathcal {F}_\xi (H, u)^t J Du + (Du)^t J D\mathcal {F}_\xi (H, u) \Big ] \,. \end{aligned}$$

Since *L*[*u*] has zero average on *M* (by its definition and integration by parts), the estimates on the Sobolev norms of *L*[*u*] follow from the above equation and Theorem 3.1. In fact, the second part of the theorem is equivalent to the following a priori estimate: for $s>s_0$ and $t<s-s_0$ there exists $C_{s,t}(\xi )>0$ such that, for all $v \in H^{s+1}_\omega (M)$ of zero average,

2 $$\begin{aligned} \Vert v \Vert _{H^t_\omega (M)} \le C_{s,t}(\xi ) \Vert X_\xi v \Vert _{H^s_\omega (M)}. \end{aligned}$$

The above a priori estimate can be derived from Theorem 3.1 as follows. For any $v \in H^\infty _\omega (M) = \cap _{s>0} H^s_\omega (M)$, the function $X_\xi v \in \text {ker}(\mathcal {I}^s_\xi (M))$ for all s>0. From Theorem 3.1 it follows the the a priori estimate (2) holds. The a priori estimate can then be extended by continuity, since $H^\infty _\omega (M)$ is dense in $H^s_\omega (M)$ for all s>0. □

## Para-Linearization

We proceed to compute the para-linearization of the non-linear equation of an invariant surface.

### Lemma 4.1

(see [LGJV05], Lemma 20). The following identities for the linearization of $\mathcal {F}_\xi (H, u)$ hold:

$$\begin{aligned} D_u \mathcal {F}_\xi (H, u) (M[u] v) = M[u] \begin{pmatrix} 0_2 & S[u] \\ 0_2 & 0_2 \end{pmatrix} v - M[u] X_\xi v + B[u,L[u],\mathcal {F}_\xi (H, u)] v. \end{aligned}$$

In the above formula we have

$$\begin{aligned} S[u]:= (I_2 + (N[u] L[u])^2)^{-1} N[u] \cdot (Du)^t \cdot [A[u], J] \cdot Du \cdot N[u] \in M_{2\times 2}({ \mathbb {R}}) . \end{aligned}$$

Finally, the following crucial property holds: the term $B[u, L[u], \mathcal {F}_\xi (H, u)]$ is rational in *L*[*u*], linear in $\mathcal {F}_\xi (H, u)$, and vanishes for $L[u] = \mathcal {F}_\xi (H, u)=0$, in fact

$$\begin{aligned} B [u, L[u], \mathcal {F}_\xi (H, u)] = \begin{pmatrix} B_1[\mathcal {F}_\xi (H, u)]&B_2[u, L[u], \mathcal {F}_\xi (H, u)] \end{pmatrix} \end{aligned}$$

where for any mapping $L, E: M \rightarrow M_{2\times 2}({ \mathbb {R}})$ we have

$$\begin{aligned} \begin{aligned} B_1[E]&:= DE , \\ B_2[u,L, E]&:= (Du) (I_2 + (N[u] L)^2)^{-1} \\ &\qquad \times \Big \{N[u]L N[u] [DE^t \cdot Du \cdot N[u] + N[u] (Du)^t J DE \cdot N[u] - N[u]L X_\xi N[u] \Big \} \\ &+ J (Du) N[u] (I_2 + (L N[u])^2)^{-1} \\&\qquad \times \Big \{ -DE^t \cdot Du \cdot N[u] + L N[u] (Du)^t [A[u], J] Du \cdot N[u] \\ &\qquad \qquad + L N[u] (Du)^t J D\mathcal {F}_\xi (H, u) \cdot N[u] - LN[u]L X_\xi N[u] \Big \} . \end{aligned} \end{aligned}$$

We may then rewrite the above relation in the equivalent form

$$\begin{aligned} &  D_u \mathcal {F}_\xi (H, u) (v) = M[u] \begin{pmatrix} 0_2 & S[u] \\ 0_2 & 0_2 \end{pmatrix} M[u]^{-1} v - M[u] X_\xi (M[u]^{-1} v) \\ &  \quad + B[u,L[u], \mathcal {F}_\xi (H, u) ] M[u]^{-1}v. \end{aligned}$$

### Proof

Let $\mathcal {F}_\xi (H, u):= X_H \circ u - X_\xi (u)$. It follows that

$$\begin{aligned} D_u \mathcal {F}_\xi (H, u) ( M[u] v) = A[u] (M[u] v) - (X_\xi M[u] ) v - M[u] (X_\xi v). \end{aligned}$$

We compute the matrix $A[u] M[u] - X_\xi M[u]$. We compute the first two columns. Since for *M*[*u*] they are given by *Du* and we have

$$\begin{aligned} D\mathcal {F}_\xi (H, u):= A[u] Du - X_\xi (Du), \end{aligned}$$

it follows that the first two columns of $A[u] M[u] - X_\xi M[u]$ are equal to $D\mathcal {F}_\xi (H, u)$, hence $B_1[E]= DE$ as stated.

We then compute the last two columns. The last two columns for *M*[*u*] are (JDu)·N[u]. We therefore compute

$$\begin{aligned} A[u] \cdot J Du \cdot N[u] - X_\xi (JDu \cdot N[u]). \end{aligned}$$

Since *Du* and *JDu* form a basis of ${ \mathbb {R}}^2$ and *N*[*u*] is invertible (for *u* near $u_0$) there exist matrices *P* and $Q \in M_{2\times 2}({ \mathbb {R}})$ such that we can write

$$\begin{aligned} A[u] \cdot J Du \cdot N[u] - X_\xi (JDu \cdot N[u]) = (Du) P + J (Du) N[u] Q . \end{aligned}$$

Since $$ (Du)^t J Du = L[u]$$, $$(Du)^t (Du) N =I_2$$, $$J^2 =-I_4$$ and $$ J A[u] = - A[u]^t J$$, we have

$$\begin{aligned} -L[u] P + Q&= -Du^t J \Big ( A[u] \cdot J Du \cdot N[u] - X_\xi (J Du \cdot N[u]) \Big ) \\&= - Du^t (A[u])^t Du \cdot N[u] - Du^t X_\xi (Du \cdot N[u]) \\&= - \Big ( X_\xi (Du)^t + [D\mathcal {F}_\xi (H, u)]^t \Big ) \cdot Du \cdot N[u] - (Du)^t X_\xi (Du \cdot N[u]) \\&= - X_\xi \Big ( (Du)^t \cdot Du \cdot N[u] \Big ) - [D\mathcal {F}_\xi (H, u)]^t \cdot Du \cdot N[u] \\&= - [D\mathcal {F}_\xi (H, u)]^t \cdot Du \cdot N[u]\,. \end{aligned}$$

We also have, since $$ (Du)^t J Du =L[u]$$,

$$\begin{aligned}&P + N[u] L[u] N[u] Q= N[u] (Du)^t \Big ( A[u] \cdot J Du \cdot N[u] - X_\xi (J Du \cdot N[u]) \Big ) \\&\quad = N[u] (Du)^t \Big ( A[u] \cdot J Du \cdot N[u] - X_\xi (J Du) \cdot N[u] \Big ) - N[u]L[u] X_\xi N[u] \\&\quad = N[u] (Du)^t \Big ( A[u] \cdot J Du \cdot N[u] - J \Big ( A[u](Du)- D\mathcal {F}_\xi (H, u) \Big ) \cdot N[u] \Big ) - N[u]L[u] X_\xi N[u] \\&\quad = N[u] (Du)^t [A[u], J] Du \cdot N[u] + N[u] (Du)^t J D\mathcal {F}_\xi (H, u) \cdot N[u] - N[u]L[u] X_\xi N[u] \,. \end{aligned}$$

Thus we have the formulas

$$\begin{aligned} {\left\{ \begin{array}{ll} P & = (I_2 + (N[u] L[u])^2)^{-1}\Big \{N[u]L[u] N[u] [D\mathcal {F}_\xi (H, u)]^t \cdot Du \cdot N[u] \\  & + N[u] (Du)^t [A[u], J] Du \cdot N[u] + N[u] (Du)^t J D\mathcal {F}_\xi (H, u) \cdot N[u] - N[u]L[u] X_\xi N[u] \Big \} ; \\ Q & = (I_2 + (L[u] N[u])^2)^{-1}\Big \{-D\mathcal {F}_\xi (H, u)^t \cdot Du \cdot N[u] + L[u] N[u] (Du)^t [A[u], J] Du \cdot N[u] \\  & \quad + L[u] N[u] (Du)^t J D\mathcal {F}_\xi (H, u) \cdot N[u] - L[u] N[u]L[u] X_\xi N[u] \Big \} . \end{array}\right. } \end{aligned}$$

We conclude that the stated identity holds with

$$\begin{aligned} \begin{aligned}&S[u]:= (I_2 + (N[u] L[u])^2)^{-1} N[u] \cdot (Du)^t \cdot [A[u], J] \cdot Du \cdot N[u] \} , \\ B_2[u]:=&(Du) (I_2 + (N[u] L[u])^2)^{-1}\Big \{N[u]L[u] N[u] [D\mathcal {F}_\xi (H, u)]^t \cdot Du \cdot N[u] \\ &+ N[u] (Du)^t J D\mathcal {F}_\xi (H, u) \cdot N[u] - N[u]L[u] X_\xi N[u] \Big \} \\ &+ J (Du) N[u] (I_2 + (L[u] N[u])^2)^{-1} \Big \{ -D\mathcal {F}_\xi (H, u)^t \cdot Du \cdot N[u] \\ &+ L[u] N[u] (Du)^t [A[u], J] Du \cdot N[u] + L[u] N[u] (Du)^t J D\mathcal {F}_\xi (H, u) \cdot N[u] \\ &- L[u]N[u]L[u] X_\xi N[u] \Big \} . \end{aligned} \end{aligned}$$

□

The para-linearization formula of Proposition 2.4 gives

$$\begin{aligned} \mathcal {F}_\xi (H, u) = \mathcal {F}_\xi (H, u_0) + T_{D_u \mathcal {F}_\xi (H, u)} (u-u_0) + \mathcal {R}_{PL} (\mathcal {F}_\xi (H, u), u-u_0) (u-u_0). \end{aligned}$$

Let then

$$\begin{aligned} E:= \mathcal {F}_\xi (H, u) . \end{aligned}$$

By the above lemma we have

$$\begin{aligned} \begin{aligned}&T_{D_u \mathcal {F}_\xi (H, u)} (u-u_0) = Op \Big ( M[u] \begin{pmatrix} 0_2 & S[u] \\ 0_2 & 0_2 \end{pmatrix} M[u]^{-1} \Big ) (u-u_0) \\ &- T_{ M[u] } X_\xi \Big (T_{M[u]^{-1} } (u-u_0) \Big ) + Op \Big ( B[u,L, E] (M[u])^{-1} \Big ) (u-u_0) + \mathcal {R}'_{CM}[u] (u-u_0) , \end{aligned} \end{aligned}$$

hence the para-linearization formula can be written as follows:

$$\begin{aligned} \begin{aligned} E&= \mathcal {F}_\xi (H, u_0) + T_{M[u]} \begin{pmatrix} 0_2 & T_{S[u]} \\ 0_2 & 0_2 \end{pmatrix} T_{M[u]^{-1}} (u-u_0) \\ &\quad - T_{ M[u] } X_\xi \Big (T_{M[u]^{-1} } (u-u_0) \Big ) + Op \Big ( B [u,E] (M[u])^{-1} \Big ) (u-u_0) \\ &\quad + \mathcal {R}_{PL}[E, u-u_0 ] (u-u_0) + \mathcal {R}_{CM}[u] (u-u_0) . \end{aligned} \end{aligned}$$

The above formula leads to the para-differential (co)homological equation

$$\begin{aligned} \begin{aligned}&T_{M[u]} \begin{pmatrix} 0_2 & T_{S[u]} \\ 0_2 & 0_2 \end{pmatrix} T_{M[u]^{-1}} (u-u_0) - T_{ M[u] } X_\xi \Big (T_{M[u]^{-1} } (u-u_0) \Big ) \\ &\quad = -\mathcal {F}_\xi (H, u_0) -\mathcal {R}_{PL}[E, u-u_0 ] (u-u_0) - \mathcal {R}_{CM}[u] (u-u_0) . \end{aligned} \end{aligned}$$

## Solution of the Para-Differential Cohomological Equation

We write the cohomological equation with counter-terms. Let $\{\chi _i\}$ be a dual basis of the space of invariant distributions for the vector field $X_\xi$. Let $[\chi _i]$ denote the 4×2 matrix with entries all equal to $\chi _i$ and $c_i$ a constant diagonal 4×4 matrix. Let $\mathcal {T}[u]$ denote a bounded linear operator (to be determined) and consider the following para-cohomological equation with counter term:

3 $$\begin{aligned} \begin{aligned}&T_{M[u]} \begin{pmatrix} 0_2 & T_{S[u]} \\ 0_2 & 0_2 \end{pmatrix} T_{M[u]^{-1}} (u-u_0) - T_{ M[u] } X_\xi \Big (T_{M[u]^{-1} } (u-u_0) \Big ) + \mathcal {T}[u] \Big ( \sum _i c_i [\chi _i] \Big ) \\ &\qquad = -\mathcal {F}_\xi (H, u_0) -\mathcal {R}_{PL}[E, u-u_0 ] (u-u_0) - \mathcal {R}_{CM}[u] (u-u_0) \,. \end{aligned} \end{aligned}$$

At this point we prove existence of solutions (that is, invertibility of the operator) for the equation

$$\begin{aligned} T_{M[u]} \begin{pmatrix} 0_2 & T_{S[u]} \\ 0_2 & 0_2 \end{pmatrix} T_{M[u]^{-1}} v - T_{ M[u] } X_\xi \Big (T_{M[u]^{-1} } v \Big ) + \mathcal {T}[u] \Big ( \sum _i c_i [\chi _i] \Big ) = f \end{aligned}$$

Since $M[u]= \text {diag}(I_2, -I_2) + O( \Vert u -u_0 \Vert _{C^1_\omega (M)})$, it is an invertible matrix for $\Vert u -u_0 \Vert _{C^1_\omega (M)}$ small enough. Under this hypothesis we can write the equation in the form

$$\begin{aligned} \begin{pmatrix} 0_2 & T_{S[u]} \\ 0_2 & 0_2 \end{pmatrix} T_{M[u]^{-1}} v - X_\xi \Big (T_{M[u]^{-1} } v \Big ) + T_{M[u]}^{-1} \mathcal {T}[u] \Big ( \sum _i c_i [\chi _i] \Big ) = T_{M[u]}^{-1} f \end{aligned}$$

hence with the choice of the linear operator $\mathcal {T}[u] = T_{M[u]}$ we have the equation

$$\begin{aligned} \begin{pmatrix} 0_2 & T_{S[u]} \\ 0_2 & 0_2 \end{pmatrix} T_{M[u]^{-1}} v - X_\xi \Big (T_{M[u]^{-1} } v \Big ) + \sum _i c_i [\chi _i] = T_{M[u]}^{-1} f \end{aligned}$$

The existence (with bounds) of solution to the above equation is proved below.

### Lemma 5.1

Let $\xi \in { \mathbb {R}}^2 \setminus \{0\}$ be such that the translation vector field $X_\xi$ on the translation surface (M,ω) is stable (that is, the Lie derivative operator has closed range) of finite codimension in Sobolev spaces of finitely differentiable functions, with loss of σ>0 derivatives in the scale $\{H^s_\omega (M) \}$ of weighted Sobolev spaces on (M,ω). There are constants $\rho _1$, $\rho _2>0$ depending on $\Vert H \Vert _{C^3_\omega (M)}$ and a constant K>0 with the following property. If $\Vert \mathcal {F} (H, u_0)\Vert _{C^1_\omega (M)} \le \rho _1$, and the embedding $u: M \rightarrow M \times { \mathbb {R}}^2$ is such that $u\vert \Sigma = u_0 \vert \Sigma = \text {Id}_\Sigma$ and $\Vert u- u_0\Vert _{C^1_\omega (M)} \le \rho _2$, then the linear para-homological equation in the unknown (*v*, *c*),

$$\begin{aligned} T_{M[u]} \begin{pmatrix} 0_2 & T_{S[u]} \\ 0_2 & 0_2 \end{pmatrix} T_{M[u]^{-1}} v - T_{ M[u] } X_\xi \Big (T_{M[u]^{-1} } v \Big ) + T_{M[u]}\Big ( \sum _i c_i [\chi _i] \Big ) = f , \end{aligned}$$

has a linear solution operator

$$\begin{aligned} (v, c) = (v_1, v_2, c_1, c_2) = (\mathcal {L}[u] (f), P[u] (f)) =(\mathcal {L}_1[u] (f), \mathcal {L}_2[u] (f), P_1[u] (f), P_2[u] (f) ) \end{aligned}$$

such that the range of the operator $\mathcal {L}[u]$ is contained in the subspace of functions vanishing at Σ, the range of the operator *P* is finite dimensional, and the following estimate holds: for a function Φ increasing in all of its arguments, we have

$$\begin{aligned} \Vert \mathcal {L} [u] (f) \Vert _{H^s_\omega (M)} + \vert P[u] (f) \vert \le C_s (K, \Vert H\Vert _{C^3_\omega (M)}, \xi ) \Vert f \Vert _{H^{s+2\sigma }_\omega (M)} . \end{aligned}$$

Moreover, the four linear operators of concern are all continuously differentiable mappings from $u\in C^1_\omega (M)$ to the space of linear operators (with operator norm).

### Proof

We remark that by the definition of the matrix *S*[*u*] we have

$$\begin{aligned} \Vert S[u] +I_2\Vert = \Vert S[u] - S[u_0] \Vert \le C \Vert H \Vert _{C^3_\omega (M)} \Vert u-u_0 \Vert _{C^1_\omega (M)} . \end{aligned}$$

In fact, in our case we have that,

$$\begin{aligned} A[u_0] = \begin{pmatrix} 0_2 & I_2 \\ 0_2 & 0_2 \end{pmatrix} \end{aligned}$$

and $$Du_0^t =\begin{pmatrix} I_2&0_2 \end{pmatrix}$$, hence $L[u_0] =0$ and thus

$$\begin{aligned} S[u_0] = \begin{pmatrix} I_2&0_2 \end{pmatrix} \begin{pmatrix} -I_2 & 0_2 \\ 0_2 & I_2 \end{pmatrix} \begin{pmatrix} I_2 \\ 0_2 \end{pmatrix} = -I_2. \end{aligned}$$

There exists $\rho _2>0$ such that for $\Vert u-u_0 \Vert _{C^1_\omega (M)} \le \rho _2$, the para-products $T_{M[u]}$ and $T_{M[u]^{-1}}$ are invertible with inverse such that

$$\begin{aligned} \Vert T^{-1}_{M[u]} - \text {diag} (I_2, -I_2) \Vert _{H^s_\omega (M)} + \Vert T^{-1}_{M[u]^{-1}} - \text {diag} (I_2, -I_2) \Vert _{H^s_\omega (M)} \le 1/2. \end{aligned}$$

The equation can then be written as a system of two systems of 2 equations equations for the unknown vector-valued function $\hat{v}:= T_{M[u]^{-1}} v$:

$$\begin{aligned} {\left\{ \begin{array}{ll} T_{S[u]} \hat{v}_2 - X_\xi \hat{v}_1 + \sum _i c_{i,1} {[}\chi _{i}]_1 = (T^{-1}_{M[u]} f )_1 , \\ \quad \quad \quad - X_\xi \hat{v}_2 + \sum _i c_{i,2} [\chi _{i}]_2 = (T^{-1}_{M[u]} f )_2, \end{array}\right. } \end{aligned}$$

This system can be solved by first solving the second equation, for an appropriate choice of the constants $\{c_{i,2}\}$. The solution $\hat{v}_2$ which unique up to additive constants, can be plugged in the first equation, which can then be solved for an appropriate choice of the constants $\{c_{i,1}\}$. More precisely the equation

$$\begin{aligned} - X_\xi \hat{v}_2 + \sum _i c_{i,2} [\chi _{i}]_2 = (T^{-1}_{M[u]} f )_2 \end{aligned}$$

can be solved in $H^{s+\sigma }_\omega (M)$ if (and only if) $c_{i,2} = D_i \Big ( (T^{-1}_{M[u]} f )_2\Big )$, for all invariant distributions $D_i$ in $H^{-(s+ 2\sigma )}_\omega (M)$, hence there exists a constant $C_{2,s}(\xi ) >0$ such that

$$\begin{aligned} \sum _i \vert c_{i,2} \vert \le C_{2,s} (\xi ) \Vert u \Vert _{C^1_\omega (M)} \Vert f \Vert _{H^{s+2\sigma }_\omega (M)}. \end{aligned}$$

The solution satisfies the estimate

$$\begin{aligned} \begin{aligned} \Vert v_2 \Vert _{H^{s + \sigma }_\omega (M)}&\le C'_{2,s} (\xi ) \Vert (T^{-1}_{M[u]} f )_2- \sum _i c_{i,2} [\chi _{i}]_2\Vert _{H^{s + 2\sigma }_\omega (M)} \\ &\le C''_{2,s} (\xi ) \Vert u \Vert _{C^1_\omega (M)} \Vert f \Vert _{H^{s + 2\sigma }_\omega (M)} . \end{aligned} \end{aligned}$$

The first equation can be then solved in $H^s_\omega (M)$ if

$$\begin{aligned} c_{i,1} = D_i \Big ( (T^{-1}_{M[u]} f )_1 - T_{S[u]} \hat{v}_2\Big ), \end{aligned}$$

for all invariant distributions $D_i$ in $H^{-(s+ \sigma )}_\omega (M)$, hence there exists a constant $C_{1,s}(\xi ) >0$ such that

$$\begin{aligned} \sum _i \vert c_{i,2} \vert \le \Vert (T^{-1}_{M[u]} f )_1 - T_{S[u]} \hat{v}_2 \Vert _{H^{s+\sigma }_\omega (M)} \le C_{1,s} (\xi ) \Vert u \Vert _{C^1_\omega (M)} \Vert f \Vert _{H^{s+2\sigma }_\omega (M)}. \end{aligned}$$

The solution of the first equation then satisfies the bound

$$\begin{aligned} \begin{aligned} \Vert v_1 \Vert _{H^{s}_\omega (M)}&\le C'_{2,s} (\xi ) \Vert (T^{-1}_{M[u]} f )_1 - T_{S[u]} \hat{v}_2- \sum _i c_{i,1} [\chi _{i}]_1\Vert _{H^{s + \sigma }_\omega (M)} \\ &\le C''_{2,s} (\xi ) \Vert u \Vert _{C^1_\omega (M)} \Vert f \Vert _{H^{s + 2\sigma }_\omega (M)} . \end{aligned} \end{aligned}$$

Finally, continuous differentiability of the operators for $u\in C^1_\omega (M)$ follows immediately from properties of para-products, that is, continuous differentiability of $T_a$ in *a* and $T_{ab}- T_aT_ b$ with respect to $(a, b)\in (L^{\infty })^2$. □

## Conclusion and Open Questions

In this section we proceed to prove the main theorem.

### Proof of Theorem 1.1

Let $N_s$ be the smallest integer >2s-1. Let $H \in C^{N_s+4}_\omega (M)$ so that the Hamiltonian vector field $X_H$ has coefficients in $C^{N_s+3}_\omega (M)$. We can assume $\Vert u \Vert _{H^s_\omega (M)} \le 1$, that $\rho _1$, $\rho _2>0$ are as in Lemma 5.1 and also assume that the hypotheses of Lemma 5.1 are satisfied, that is

4 $$\begin{aligned} \Vert u \Vert _{H^s_\omega (M)} \le 1\,, \quad \Vert \mathcal {F} (H, u_0) \Vert _{C^1_\omega (M)} \le \rho _1\,, \quad \Vert u-u_0\Vert _{C^1_\omega (M)} \le \rho _2 \,. \end{aligned}$$

The para-cohomological equation has the form

$$\begin{aligned} u-u_0 = - \mathcal {L}[u] \Bigl (\mathcal {F}_\xi (H, u_0) + \mathcal {R}_{PL}[\mathcal {F} (H, u), u-u_0 ] (u-u_0) + \mathcal {R}_{CM}[u] (u-u_0) \Big ) \end{aligned}$$

By Lemma 5.1 we have

$$\begin{aligned} \Vert \mathcal {L}[u] \Bigl (\mathcal {F}_\xi (H, u_0) \Big ) \Vert _{H^s_\omega (M)} \le C_s (K, \Vert H\Vert _{C^3_\omega (M)}, \xi ) \Vert \mathcal {F}_\xi (H, u_0) \Vert _{H^{s+2\sigma }_\omega (M)} \end{aligned}$$

We also have, for 2t-1>s+2σ,

$$\begin{aligned} \begin{aligned} \Vert \mathcal {L}[u]&\Bigl ( \mathcal {R}_{PL}[\mathcal {F} (H, u), u-u_0 ] (u-u_0) \Big )\Vert _{H^s_\omega (M)} \\ &\quad \le C_s (K, \Vert H\Vert _{C^3_\omega (M)}, \xi ) \Vert \mathcal {R}_{PL}[\mathcal {F} (H, u), u-u_0 ] (u-u_0) \Vert _{H^{s+ \sigma }_\omega (M)} \\ &\quad \le C_s (K, \Vert H\Vert _{C^3_\omega (M)}, \xi ) C_s \Big ( \Vert \mathcal {F}_\xi (H, u_0) \Vert _{H^{s+2\sigma }_\omega (M)} \Vert u-u_0 \Vert _{H^t_\omega (M)} \\ &\qquad \qquad \qquad \qquad \qquad \qquad \qquad + \Vert H \Vert _{C^{N_s+4}_\omega (M)} \Vert u-u_0 \Vert _{H^t_\omega (M)} ^2\Big ). \end{aligned} \end{aligned}$$

The regularizing remainder $\mathcal {R}_{CM}[u] (u-u_0)$ is equal to the expression

$$\begin{aligned} \begin{aligned} \mathcal {R}_{CM}[u] (u-u_0)&= Op \Big ( M[u] \begin{pmatrix} 0_2 & S[u] \\ 0_2 & 0_2 \end{pmatrix} M[u]^{-1} \Big ) \\ &- Op\big (M[u] X_\xi M[u]^{-1} \Big ) - T_{M[u]} \begin{pmatrix} 0_2 & T_{S[u]} \\ 0_2 & 0_2 \end{pmatrix} T_{M[u]^{-1}} + T_{M[u]} X_\xi T_{M[u]^{-1}}. \end{aligned} \end{aligned}$$

Since for s>t>1+(s+σ)/2 we have that $\Vert S[u] \Vert _{ C_\omega ^{s+ \sigma -t}(M) }$ is bounded by an increasing function of $\Vert H \Vert _{ H^{N_s}_\omega (M)}$ and $\Vert u-u_0 \Vert _{ H^t_\omega (M)}$, and in addition

$$\begin{aligned} \Vert M[u] - \text {diag} (I_2, -I_2) \Vert _{ C_\omega ^{s+ \sigma -t}(M) } \le \Vert u-u_0 \Vert _{ H^t_\omega (M)}, \end{aligned}$$

we derive the estimate

$$\begin{aligned} \Vert \mathcal {R}_{CM}[u] (u-u_0) \Vert _{ H^{s+ \sigma }_\omega (M)} \le C'_s \Vert u- u_0 \Vert ^2_ { H^t_\omega (M)} , \end{aligned}$$

which in turn implies by Lemma 5.1 that

$$\begin{aligned} \begin{aligned} \Vert \mathcal {L}[u] \Bigl ( \mathcal {R}_{CM}[u] (u-u_0) \Big )\Vert _{H^s_\omega (M)}&\le C_s (K, \Vert H\Vert _{C^3_\omega (M)}, \xi ) \Vert \mathcal {R}_{CM}[u] (u-u_0)\Vert _{H^{s+ \sigma }_\omega (M)} \\ &\le C_s (K, \Vert H\Vert _{C^3_\omega (M)}, \xi ) C'_s \Vert u- u_0 \Vert ^2_ { H^t_\omega (M)}. \end{aligned} \end{aligned}$$

We then argue that under conditions (4), if $\Vert \mathcal {F}_\xi (H, u_0) \Vert _{H^{s+2\sigma }_\omega (M)}$ is sufficiently small, there exists ρ>0 such that if $\Vert u- u_0 \Vert _ { H^t_\omega (M)} \le \rho$, then

$$\begin{aligned} \begin{aligned} C_s&(K, \Vert H\Vert _{C^3_\omega (M)}, \xi ) \Vert \mathcal {F}_\xi (H, u_0) \Vert _{H^{s+2\sigma }_\omega (M)} \\ &+ C_s (K, \Vert H\Vert _{C^3_\omega (M)}, \xi ) C_s \Big ( \Vert \mathcal {F}_\xi (H, u_0) \Vert _{H^{s+2\sigma }_\omega (M)} \rho + \Vert H \Vert _{C^{N_s+4}_\omega (M)} \rho ^2\Big ) \\ &\qquad \qquad + C_s (K, \Vert H\Vert _{C^3_\omega (M)}, \xi ) C'_s \rho ^2 \le \rho . \end{aligned} \end{aligned}$$

Let then $\mathcal {S}$ denote the operator defined for all functions $u\in B_{H^t_\omega (M)} (u_0, \rho )$, that is, such that $u \vert \Sigma = u_0 \vert \Sigma = \text {Id}_\Sigma$ and $\Vert u -u_0 \Vert _{H^t_\omega (M)} < \rho$ as

$$\begin{aligned} \mathcal {S}(u):= u_0 - \mathcal {L}[u] \Bigl (\mathcal {F}_\xi (H, u_0) + \mathcal {R}_{PL}[\mathcal {F} (H, u), u-u_0 ] (u-u_0) + \mathcal {R}_{CM}[u] (u-u_0) \Big ). \end{aligned}$$

We have proved above that $\mathcal {S}: B_{H^t_\omega (M)} (u_0, \rho ) \rightarrow B_{H^s_\omega (M)} (u_0, \rho )$ with s>t, and since the embedding $H^s_\omega (M)$ into $H^t_\omega (M)$ is compact, by the Schauder fixed point theorem the operator $\mathcal {S}$ has a fixed point $u \in B_{H^t_\omega (M)} (u_0, \rho )$. In fact, for sufficiently small ρ>0 it is possible to apply the inverse function theorem in the Hilbert space $H^s_\omega (M)$, hence the fixed point is unique and depends smoothly with respect to the Hamiltonian *H*. In addition, there exists a constant $C(\xi , \Vert H \Vert _{C^{N_s +4}_\omega (M)}) >0$ such that

5 $$\begin{aligned} \Vert u- u_0 \Vert _{H^t_\omega (M)} \le C(\xi , \Vert H \Vert _{C^{N_s +4}_\omega (M)}) \cdot \Vert \mathcal {F}_\xi (H, u_0) \Vert _{H^{s+2\sigma }_\omega (M)} \,. \end{aligned}$$

For the fixed point we have the identity

$$\begin{aligned} \mathcal {F}_\xi (H, u) = Op \Big ( B[u, L[u], \mathcal {F}_\xi (H, u)] M(u)^{-1} \Big ) (u-u_0) + T_{M[u]} \Big (\sum _i P_i[u] \chi _i \Big ). \end{aligned}$$

Since the fixed point is given as a smooth function u:=u(H) of the Hamiltonian *H*, defined locally on a neighborhood of $H_0$, the condition

$$\begin{aligned} P[ u(H)] =0 , \end{aligned}$$

describes a finite codimension local submanifold of the space of Hamiltonians, as soon as we can prove that the differential of the function *P*[*u*(*H*)] (as a function of the Hamiltonian *H*) is surjective at $H=H_0$.

For Hamiltonians *H* such that P[u(H)]=0 the fixed point equation becomes

$$\begin{aligned} \mathcal {F}_\xi (H, u) = Op \Big ( B[u, L[u], \mathcal {F}_\xi (H, u)] M(u)^{-1} \Big ) (u-u_0) \end{aligned}$$

which implies $\mathcal {F}_\xi (H, u) =0$ by the contraction mapping principle (as in [AlSh]). Indeed, the right hand side can be viewed (for fixed $u\in B_{H^t_\omega (M)} (u_0, \rho )$) as a linear operator

$$\begin{aligned} \mathcal {B}_u (E):= Op \Big ( B[u, L[u], E \Big ) (u-u_0) , \quad \text { for } E \in H^t_\omega (M), \end{aligned}$$

acting on $E=\mathcal {F}_\xi (H, u)$ and vanishing for E=0 which, when applied to $\mathcal {F}_\xi (H, u)$, is a contraction of the norm of the space $H^t_\omega (M)$: there exists $\kappa \in (0,1)$ such that

$$\begin{aligned} \Vert \mathcal {B}_u \big ( \mathcal {F}_\xi (H, u) \big ) \Vert _{H^t_\omega (M)} \le \kappa \Vert \mathcal {F}_\xi (H, u) \Vert _{H^t_\omega (M)}. \end{aligned}$$

To prove that $\mathcal {B}_u$ is a contraction on $\mathcal {F}_\xi (H, u)$, we estimate by formula (5)

$$\begin{aligned} \begin{aligned}&\Vert Op \Big ( B[u,L[u], E] M(u)^{-1} \Big ) (u-u_0) \Vert _{H^t_\omega (M)} \\ &\quad \le C_s C(\xi , \Vert H \Vert _{C^{N_s+4}_\omega (M)}) \Vert \mathcal {F}_\xi (H, u_0) \Vert _{H^{s+2\sigma }_\omega (M)} \Vert B[u, L[u], E] M(u)^{-1} \Vert _{L^\infty (M)} . \end{aligned} \end{aligned}$$

By the expression of *B*(*u*, *L*, *E*) in Lemma 4.1, there exists a constant $C_B >0$ such that, under the hypothesis that $\Vert u-u_0 \Vert _{C^2_\omega (M)}$ and $\Vert L \Vert _{C^0_\omega (M)}$ are sufficiently small, we have

$$\begin{aligned} \Vert B(u,L, E) \Vert _{L^\infty (M)} \le C_B \max \Big \{ \Vert L \Vert _{C^0(M)}, \Vert E\Vert _{C^1_\omega (M)} \Big \} . \end{aligned}$$

In addition, there exists $t_0>0$ such that for $t>t_0$ and for a constant $C_t>0$ we have $\Vert E\Vert _{C^1_\omega (M)} \le C_t \Vert E\Vert _{H^t_\omega (M)}$ and, by Lemma 3.3, for a constant $C_t(\xi )>0$ we have

$$\begin{aligned} \Vert L[u] \Vert _{C^0(M)} \le C_{t}(\xi ) \Vert u \Vert _{H^t_\omega (M)} \Vert \mathcal {F}_\xi (H, u) \Vert _{H^t_\omega (M)} , \end{aligned}$$

hence, as $\Vert u \Vert _{H^t_\omega (M)}$ is bounded, there exists a constant $C'_t(\xi )>0$ such that

$$\begin{aligned} \Vert B[u,L[u], \mathcal {F}_\xi (H, u) ] M(u)^{-1} \Vert _{L^\infty (M)} \le C_t(\xi ) \Vert \mathcal {F}_\xi (H, u) \Vert _{H^t_\omega (M)}. \end{aligned}$$

Thus, if $\kappa := C_s C_t C(\xi , \Vert H \Vert _{C^{N_s+4}}) \cdot \Vert \mathcal {F}_\xi (H, u_0) \Vert _{H^{s+2\sigma }_\omega (M)} <1$ we have that $\mathcal {B}_u$ contracts the norm of $\mathcal {F}_\xi (H, u)$ in the Sobolev space $H^t_\omega (M)$ and the identity

$$\begin{aligned} (I - \mathcal {B}_u) \mathcal {F}_\xi (H, u) =0 \end{aligned}$$

indeed implies that $\mathcal {F}_\xi (H, u) =0$.

Finally we address the surjectivity of the differential at $H=H_0$ (and as a consequence $u=u_0$) of the function *P*[*u*(*H*)], which implies that the function is a local submersion, hence its zero locus is a $C^1$ submanifold. The linearization of the para-cohomological equation (3) reads

$$\begin{aligned} X_\xi v + \Big ( \sum _i d_i [\chi _i] \Big ) = - D_H\mathcal {F}_\xi (H_0, u_0) (h) . \end{aligned}$$

since $M[u_0]=\text {diag} (I_2, -I_2)$, $S[u_0]=0$, and the terms $\mathcal {R}_{PL}$, $\mathcal {R}_{CM}$ are quadratic in $u-u_0$. By Theorem 3.1 and Corollary 3.2, the above equation has a solution for the appropriate choice of the coefficients $(d_i)$ to ensure the vanishing of the obstructions. Such coefficients give the value of the differential of the map *P*[*u*(*H*)] at the tangent vector *h* representing the variation of the Hamiltonian *H* at $H_0$. It is clearly possible to find a variation *h* such that the values of the coefficients $(d_i)$ is any given vector of coefficients, as long as the obstructions are chosen to be linearly independent functionals. This argument implies that the map *P*[*u*(*H*)] is local submersion, as a function of *H*, with finite rank, hence its zero set is a local $C^1$ submanifold of finite codimension (by the implicit function theorem in Banach spaces). □

We conclude by posing a couple of natural open questions:

### Question 6.1

For translation surfaces of higher genus and non-integrable rational billiards, are there arbitrarily small smooth perturbations such that the perturbed Hamiltonian does not have any invariant surface in the homology class of the zero section, or does not have any invariant surface which is a (Lipschitz) continuous graph over the zero section ?

### Question 6.2

For translation surfaces of higher genus and non-integrable rational billiards, are there any non-trivial smooth perturbations such that the perturbed Hamiltonian has a positive measure set of the phase space foliated by invariant surfaces homologous to the zero section ? or at least an infinite number of distinct invariant surfaces homologous to the zero section ?

In the completely integrable case, by the KAM theory, the answer to the first question is negative and the answer to the second question is positive, for all sufficiently small smooth perturbations.

## Data Availability

This article has no associated data.
